# Optic Disc Pallor in Parkinson's Disease: A UK Biobank Study

**DOI:** 10.1002/mds.30127

**Published:** 2025-01-30

**Authors:** Samuel Gibbon, David P. Breen, Thomas J. MacGillivray, UK Biobank Eye & Vision Consortium

**Affiliations:** ^1^ Centre for Clinical Brain Sciences University of Edinburgh Edinburgh UK; ^2^ Robert O Curle Ophthalmology Suite Institute for Regeneration and Repair, University of Edinburgh Edinburgh UK; ^3^ Anne Rowling Regenerative Neurology Clinic University of Edinburgh Edinburgh UK; ^4^ Usher Institute of Population Health Sciences and Informatics University of Edinburgh Edinburgh UK

**Keywords:** Parkinson's disease, optic disc pallor, retina, color fundus photographs, PallorMetrics

## Abstract

**Background:**

Recent studies have suggested that retinal changes measured with optical coherence tomography are detectable in early Parkinson's disease (PD), highlighting the potential of ophthalmic biomarkers for diagnosis and monitoring.

**Objective:**

We set out to investigate the relationship between optic disc pallor measured in fundoscopy images and both prevalent and incident PD.

**Methods:**

We analyzed color fundus photographs from 787 UK Biobank participants: 89 with prevalent PD, 317 with incident PD, and 381 age‐ and sex‐matched controls. Optic disc pallor in several zones was quantified using semi‐automated software. We used logistic and linear regression, adjusted for relevant covariates, to test for associations between disc pallor and PD status and duration.

**Results:**

Participants with prevalent PD had significantly paler optic discs globally (OR per standard deviation [SD] increase = 1.39 [CI: 1.08–1.81], *P* = 0.012) and across several zones compared to controls. Each year since PD diagnosis was associated with a 1.37 SD increase in global pallor (standardized *β* = 1.37 [SE = 0.61], *P* = 0.029), and a similar increase across several zones, however, this finding was sensitive to outliers with long disease duration. No significant associations were observed for the incident PD group.

**Conclusions:**

Optic disc pallor is significantly associated with PD and may become more pronounced with disease duration. This suggests that optic disc pallor, measured in routinely taken color fundus photographs, may serve as a biomarker for PD‐related neurodegeneration. © 2025 The Author(s). *Movement Disorders* published by Wiley Periodicals LLC on behalf of International Parkinson and Movement Disorder Society.

Optical coherence tomography (OCT) enables the imaging of retinal layers and is increasingly used to assess structural changes within the eye in neurodegenerative disorders, including Parkinson's disease (PD).^1^, ^2^, ^3^, ^4^, ^5^ OCT is typically focused on one of two key landmarks in the retina: the macula or the optic disc. In the macula, the ganglion cell layer and the inner plexiform layer (GCIPL) have shown significant alterations in PD, with a recent meta‐analysis indicating a notable decrease in GCIPL thickness compared to controls.^6^ By comparison, around the optic disc, the retinal nerve fiber layer (RNFL) appears to be more affected in PD than GCIPL thickness, with a recent meta‐analysis revealing thinner peripapillary RNFL (pRNFL) in PD patients compared to controls, with the greatest thinning observed in temporal regions.^7^ The mechanisms of nerve cell loss in the eye are thought to reflect similar neurodegenerative processes occurring in the brain.^8^ In PD, the degeneration of dopaminergic neurons in the brain may extend to the retinal ganglion cells, leading to thinning of the retinal layers.^9^

Despite the imaging capabilities of OCT, there can be technical challenges for movement disorder patients due to the requirement to maintain a stable position and gaze during scan acquisition, which may take anywhere from 15 seconds to 2 minutes (depending on the machine, imaging protocol, and patient). A quicker and simpler alternative is color fundus photography, which takes only a few seconds to acquire. Furthermore, color fundus photographs are more commonly collected in the population (ie, during routine high‐street eye examinations).

Recently, we developed a new approach to examine pRNFL thickness by measuring the paleness of the neuro‐retinal rim (hereafter referred to as *optic disc pallor*) in color fundus photographs (*PallorMetrics*),^10^ under the assumption that a pale disc primarily indicates the loss or degeneration of the pRNFL.^10^, ^11^, ^12^, ^13^ One previous study has observed optic disc pallor in PD,^14^ most notably in the temporal region; however, this study was based on subjective clinical observation. By comparison, the *PallorMetrics* software generates quantitative measurements of optic disc pallor both globally and in specific zones akin to those used by modern OCT devices. *PallorMetrics* has previously been used to investigate disc pallor in relation to MRI features of cerebral small vessel disease,^15^ lacunar stroke,^16^ and myopia (manuscript under review).

In the current study, we aimed to investigate the relationship between optic disc pallor and prevalent and incident PD using data from the UK Biobank. We hypothesized that (1) optic discs would be paler in prevalent PD compared to controls, (2) pallor would become more pronounced with advancing disease duration, and (3) pallor would be detectable even prior to PD diagnosis.

## Patients and Methods

### Participants and Image Capture

The UK Biobank recruited approximately 500,000 UK‐based participants aged 40–69 between 2006 and 2010 (https://www.ukbiobank.ac.uk/). Alongside questionnaires and physical measurements, a subset of just under 70,000 participants received detailed ophthalmic assessment between 2009 and 2010, including single‐field color fundus photography (45° field‐of‐view) and OCT of each eye, both centered on the macula. Photographs and scans were captured using a digital Topcon‐1000 integrated camera (Topcon 3D OCT1000 Mark II, Topcon Corp., Tokyo, Japan). The same model device was used for all participants. The right eye was imaged first. The UK Biobank has Research Tissue Bank approval from the North‐West Research Ethics Committee (ref: 06/MRE08/75).

### Case Definition

PD diagnosis was defined as the first occurrence of PD according to ICD‐10 G20 code (any position) within NHS linked health records. In the UK Biobank, ICD‐10 codes were derived from primary care, hospital inpatient admissions, death records, and self‐reported medical conditions. Participants with a G20 code before ophthalmic assessment were defined as prevalent PD, whereas those with a G20 code after ophthalmic assessment were defined as incident PD.

### Sample Derivation and Exclusions

A total of 68,508 participants underwent color fundus photography. We selected one eye per participant for our analyses. The right eye was always chosen if available because it was captured first and therefore likely to be of better quality. In cases where the right eye photograph was not available (400 participants), the left eye was used. In cases where multiple photographs were captured for a single eye, we selected the second instance for analysis, which was usually better quality.

Based on previous findings of reduced RNFL in dementia,^3^ and in accordance with similar research,^5^ we removed participants with a diagnosis of all‐cause dementia at the time of imaging (UK Biobank Algorithmically Defined Outcome; Field ID = 42,018). Based on ICD‐10 codes, we removed participants with ocular disorders that may affect optic disc appearance, encompassing glaucoma (H40), multiple sclerosis (G35), optic neuritis (H46), visual pathway disorder (H47), blindness (H54), and retinal detachment/breaks (H33). Cataracts (H26), previous ocular surgery (self‐reported), and diabetes (E10 and E11) can also affect optic disc appearance, but their prevalence is high. Rather than excluding these participants, we opted to account for these variables statistically and perform sensitivity analyses. Disc pallor varied substantially by ethnicity (Fig. S1; Table 1). Therefore, we excluded participants with missing ethnicity data (n = 8). After exclusions, fundus photographs were available for 66,390 participants, of whom 103 individuals had a confirmed diagnosis of PD (prevalent PD), and 412 individuals later developed PD (incident PD).

**TABLE 1:** Participant demographics and study variables

	Controls (N = 381)	Prevalent (N = 89)	*P*‐value	Incident (N = 317)	*P*‐value
Age	62.7 (5.25)	61.1 (6.29)	0.033*	62.9 (5.05)	0.610
Sex (female)	148 (38.8%)	36 (40.4%)	0.874	123 (38.8%)	1.000
Ethnic category					
White	368 (95.8%)	83 (93.3%)	‐	292 (92.1%)	‐
Black	3 (0.8%)	1 (1.1%)	‐	9 (2.8%)	‐
Other/Mixed	4 (1.0%)	2 (2.2%)	‐	5 (1.6%)	‐
South Asian	6 (1.6%)	3 (3.4%)	‐	11 (3.5%)	‐
Non‐White ethnicity	13 (3.4%)	6 (6.7%)	‐	25 (7.9%)	0.015*
Hypertension	130 (34.1%)	26 (29.2%)	0.447	128 (40.4%)	0.104
Diabetes	19 (5.0%)	2 (2.2%)	‐	16 (5.0%)	1.000
Cataract	19 (5.0%)	5 (5.6%)	‐	22 (6.9%)	0.352
Eye surgery	21 (5.5%)	5 (5.6%)	‐‐	27 (8.5%)	0.158
Pallor					
Global	1.27 (0.23)	1.33 (0.25)	0.026*	1.27 (0.22)	0.869
Temporal	1.41 (0.30)	1.49 (0.31)	0.013*	1.40 (0.28)	0.935
Temporal‐inferior	1.26 (0.23)	1.32 (0.25)	0.033*	1.25 (0.22)	0.929
Nasal‐inferior	1.12 (0.19)	1.16 (0.21)	0.071	1.13 (0.18)	0.553
Nasal	1.17 (0.20)	1.22 (0.22)	0.054	1.18 (0.19)	0.416
Nasal‐superior	1.17 (0.19)	1.21 (0.22)	0.067	1.17 (0.19)	0.655
Temporal‐superior	1.25 (0.26)	1.32 (0.27)	0.029*	1.25 (0.24)	0.916
PMB	1.44 (0.31)	1.53 (0.32)	0.012*	1.43 (0.29)	0.949
NT ratio	0.84 (0.09)	0.82 (0.08)	0.042*	0.85 (0.09)	0.184
Retinal covariates					
Disc area (pixels)	25,200 (5370)	25,000 (5110)	0.636	25,400 (5870)	0.765

*Note*: All values are N (%) or mean (standard deviation). *P*‐values are for comparisons between controls vs. prevalent PD, and controls vs. incident PD. * *P*‐value <0.05; ** *P*‐value <0.01, ‐ statistical test failed to converge due to low sample size in one group.

Abbreviations: PMB, papillomacular bundle; NT ratio, nasal temporal ratio; PD, Parkinson's disease.

We then created an age‐ and sex‐matched control group using the “matchit” package in R with a ratio of 1:1, combining incident and prevalent PD, generating 515 controls. After quality control (detailed below), the final sample contained 89 individuals with prevalent PD, 317 with incident PD, and 381 controls. In effect, this meant that the ratio of prevalent PD to controls was over 4:1, and the ratio of incident PD to controls was 1.2:1.

### Optic Disc Pallor Quantification

**FIG. 1:**
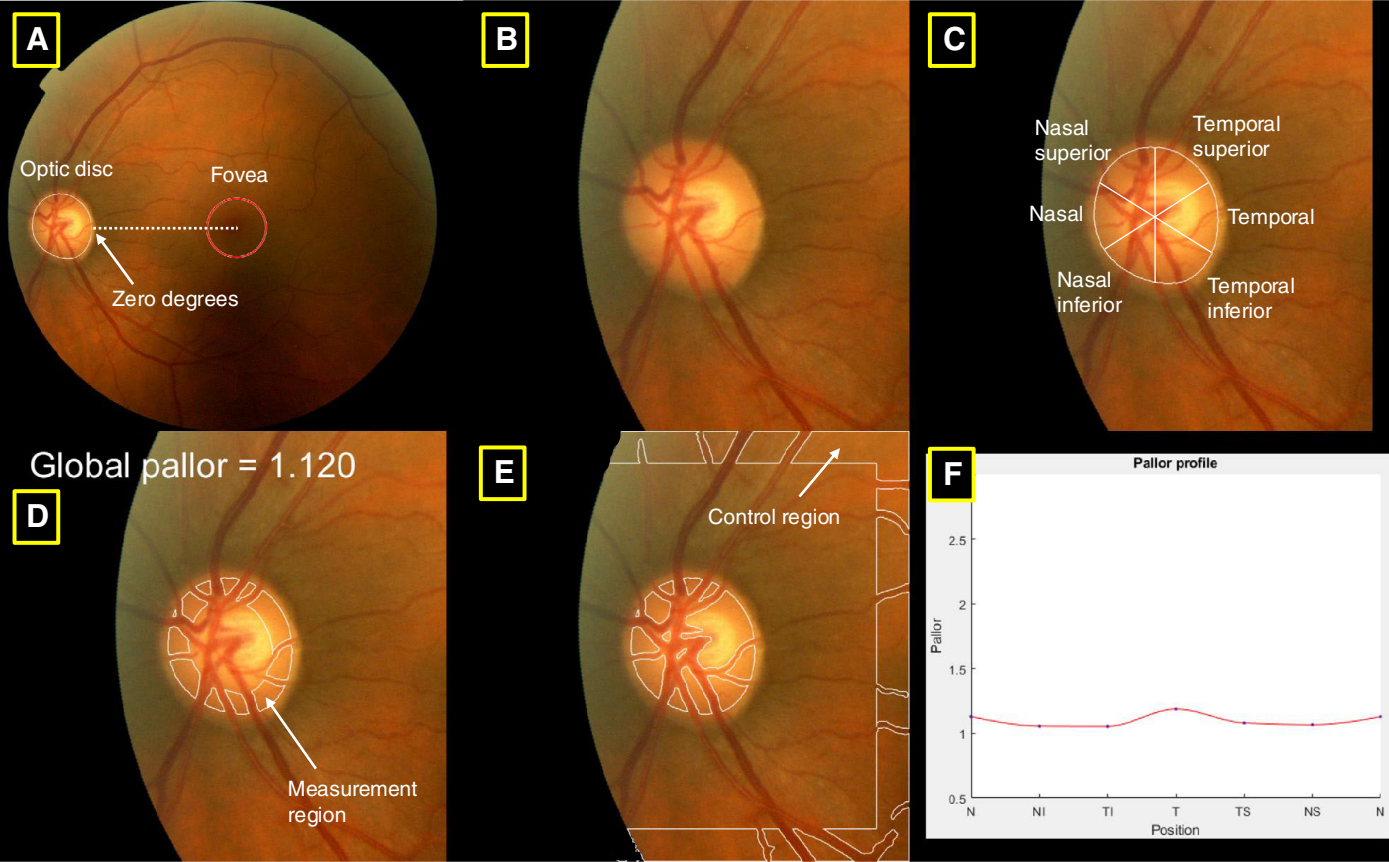
Key visualizations of *PallorMetrics* for a single image. (**A**) Image is rotated along the optic disc‐fovea axis, (**B**) cropped optic disc, (**C**) zones, (**D**) measurement region, (**E**) control region, and (**F**) optic disc pallor profile. [Color figure can be viewed at wileyonlinelibrary.com]

Optic disc pallor was measured using previously validated software, *PallorMetrics*.^10^ Briefly, a deep learning‐based approach segmented the optic disc to the inner edge of the border tissue and localized the fovea (Fig. 1A). For the current study, we used a semi‐automated version, in which we hand‐corrected the optic disc segmentation for each image by dragging the edges of a deformable ellipse (procedure detailed in Ref. 10). Although this process took longer than the fully automatic version, it minimized segmentation error. The optic disc was cropped from the image (Fig. 1B). A measurement region was then designated starting from the disc border of the cropped image, extending 30 pixels inwards (Fig. 1D). Zones were then overlaid onto this area following the standard OCT peripapillary scan pattern (Fig. 1C). The intersection of the disc‐fovea axis with the measurement region was assigned a zero‐degree value (Fig. 1A). The temporal zone extends from −45° to 45°, the temporal superior zone from −45° to −90°, and so on (Fig. 1C). The papillomacular bundle (PMB), a thick bundle of axons originating in the macula that allows for sharp central vision, is a special case of the temporal zone, extending from −15° to 15°. A control region was automatically defined by the software as starting at the outer border of the cropped optic disc image and extending 50 pixels inwards (Fig. 1E). Vessels were excluded from the measurement and control regions. Lastly, pallor was quantified by the software based on the ratio of red/green light reflectance within each zone relative to the control region. The result is a dimensionless measure of pallor (global pallor), pallor in six zones (sectoral pallor) (Fig. 1F), pallor in the PMB, and the nasal‐temporal ratio. Optic disc analyses were performed in MATLAB (version R2022b, Natick, Massachusetts: The MathWorks Inc.) within the UK Biobank's Research Analysis Platform. Key stages in the pipeline are presented in Figure 1.

### Quality Control

All images (controls, incident PD, prevalent PD) were put into a single directory, shuffled, and presented for annotation randomly with their filenames occluded. Images were rejected for one or more of the following reasons: under‐ or over‐exposure of the optic disc or control region, uneven illumination over the optic disc or control region, disc border unclear, abnormal presentation of the disc or retina, and failure to locate fovea. Example images from each rejection category are presented in Figure S2. One hundred and thirty‐one images (25.4%) were rejected from the control group, 14 images (13.6%) were rejected from the prevalent PD group, and 90 images (21.8%) were rejected from the incident PD group. Manual disc segmentation and quality control were carried out using a single annotator (author S.G.).

### Statistical Analysis and Covariates

Differences in demographics and retinal measures between the groups (control vs. prevalent PD, control vs. incident PD) were assessed using *t* tests where one variable was continuous, or *χ* ^2^ where both variables were dichotomous. We used logistic regression to estimate the odds of prevalent PD versus controls, and incident PD versus controls, with disc pallor as a predictor. The small sample size precluded the use of survival analysis (eg, Cox regression). In accordance with similar work,^5^ all models were adjusted for age, sex, ethnic category, hypertension, cataract, diabetes, previous eye surgery, and disc area. These covariates were chosen prior to analysis, and no subsequent adaptations were made. Optic disc pallor, age, and disc area were standardized prior to analysis to have a zero mean and unit variance. Self‐reported ethnicity was categorized into four groups defined by the UK Census (White, Black, Other/Mixed, South Asian); however, there were too few samples in each group, which precluded us from including ethnic category as a covariate. Instead, we used a binary ethnicity variable (White/non‐White). Only two participants with prevalent PD had diabetes; therefore, we excluded diabetes as a covariate for prevalent cases (as opposed to adjusting for this covariate or removing cases). To determine whether the duration of disease affected disc pallor, we fitted linear regression models between pallor and “years since‐ and years to diagnosis.” As before, models were adjusted for the covariates listed above. We carried out sensitivity analyses in all models by removing participants with cataracts, previous eye surgery, diabetes, and non‐White ethnicity. In the PD duration models, we also conducted a sensitivity analysis by removing “outliers” (defined as individuals with a PD duration of more than 15 years). These outliers were identified based on a visual inspection of Figure S3. Analyses were conducted in R (version 4.2.1; www. R-project.org). Statistical significance was set at *P* < 0.05.

## Results

**FIG. 2:**
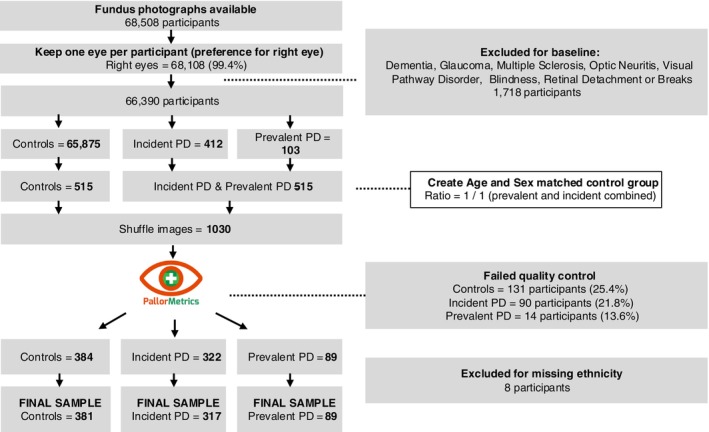
Sample derivation flowchart. [Color figure can be viewed at wileyonlinelibrary.com]

Compared with controls, those with prevalent PD were around 1.6 years younger (*P* = 0.033), and those with incident PD had a higher proportion of non‐White ethnicity (7.9% compared with 3.4%). These differences were expected given the nature of how we derived the control group (see Methods—Sample derivation and exclusions). There were no other significant differences between the groups. Optic disc pallor steadily decreased with age (Fig. S4) and varied by ethnicity (Fig. S1). Demographics and study variables are summarized in Table 1. The sample derivation flowchart is presented in Figure 2.

### Associations between Optic Disc Pallor and PD

After adjusting for covariates, and compared to the control group, participants with prevalent PD had paler optic discs globally (OR per standard deviation [SD] increase = 1.39 [CI: 1.08–1.81], *P* = 0.012) and across several zones. The point estimate was slightly higher in the temporal zone (OR per SD increase = 1.40 [CI: 1.08–1.81], *P* = 0.010) compared to the nasal zones (OR per SD increase = 1.37 for nasal zone, 1.33 for nasal‐inferior, 1.35 for nasal‐superior). The direction of association was consistent across all zones. No statistically significant associations were observed for the incident PD group; however, a trend was observed with the direction consistent across all zones, suggesting a slight increase in pallor compared with controls. In sensitivity analyses, removing participants with cataracts (N = 24) caused a slight reduction in estimates, removing participants with previous eye surgery (N = 26) slightly increased the estimates, removing participants with diabetes (N = 21) increased the estimates, and removing participants with non‐White ethnicity (N = 19) slightly reduced the estimates; all associations bar one (nasal‐superior pallor in the sensitivity to cataract analysis) remained statistically significant. Results are summarized in Table 2 and plotted in Fig. S5.

**TABLE 2:** Logistic regression results comparing prevalent PD and incident PD to controls

	Prevalent PD			Incident PD		
Pallor	1 SD	Odds ratio per SD increase (CI)	*P* value	1 SD	Odds ratio per SD increase (CI)	*P* value
Global	0.24	1.39 (1.08–1.81)	0.012*	0.23	1.06 (0.90–1.25)	0.490
Temporal	0.30	1.40 (1.08–1.81)	0.010*	0.29	1.04 (0.88–1.22)	0.680
Temporal‐inferior	0.23	1.38 (1.07–1.78)	0.014*	0.23	1.03 (0.87–1.22)	0.717
Nasal‐inferior	0.19	1.33 (1.04–1.71)	0.025*	0.18	1.10 (0.93–1.29)	0.262
Nasal	0.20	1.37 (1.06–1.77)	0.015*	0.19	1.12 (0.95–1.32)	0.169
Nasal‐superior	0.20	1.35 (1.04–1.74)	0.022*	0.19	1.09 (0.92–1.28)	0.322
Temporal‐superior	0.25	1.38 (1.07–1.78)	0.014*	0.24	1.05 (0.89–1.23)	0.582
PMB	0.31	1.40 (1.08–1.81)	0.011*	0.30	1.04 (0.88–1.22)	0.664
NT ratio	0.09	0.83 (0.65–1.06)	0.141	0.09	1.10 (0.94–1.28)	0.231
Sensitivity analysis						
Without cataract (N = 24 removed)						
Global	0.24	1.36 (1.04–1.78)	0.023*			
Temporal	0.30	1.37 (1.05–1.78)	0.019*			
Temporal‐inferior	0.24	1.36 (1.05–1.78)	0.021*			
Nasal‐inferior	0.19	1.28 (0.99–1.65)	0.060			
Nasal	0.20	1.33 (1.03–1.74)	0.030*			
Nasal‐superior	0.20	1.32 (1.02–1.72)	0.037*			
Temporal‐superior	0.26	1.34 (1.03–1.74)	0.028*			
PMB	0.31	1.37 (1.06–1.79)	0.019*			
NT ratio	0.09	0.83 (0.64–1.06)	0.140			
Without eye surgery (N = 26 removed)						
Global	0.24	1.41 (1.08–0.32)	0.011*			
Temporal	0.30	1.41 (1.09–1.84)	0.009**			
Temporal‐inferior	0.23	1.40 (1.08–1.83)	0.011*			
Nasal‐inferior	0.19	1.34 (1.04–1.74)	0.024*			
Nasal	0.20	1.38 (1.06–1.79)	0.016*			
Nasal‐superior	0.20	1.36 (1.05–1.76)	0.022*			
Temporal‐superior	0.25	1.39 (1.07–1.80)	0.013*			
PMB	0.31	1.41 (1.09–1.84)	0.010*			
NT ratio	0.09	0.82 (0.64–1.05)	0.120			
Without diabetes (N = 21 removed)						
Global	0.24	1.45 (1.12–1.90)	0.005**			
Temporal	0.30	1.44 (1.11–1.88)	0.006**			
Temporal‐inferior	0.24	1.43 (1.10–1.86)	0.008**			
Nasal‐inferior	0.19	1.40 (1.09–1.81)	0.009**			
Nasal	0.20	1.44 (1.12–1.87)	0.005**			
Nasal‐superior	0.20	1.40 (1.08–1.82)	0.010*			
Temporal‐superior	0.26	1.43 (1.11–1.86)	0.007**			
PMB	0.31	1.44 (1.11–1.87)	0.006**			
NT ratio	0.08	0.85 (0.67–1.09)	0.208			
White ethnicity only (N = 19 removed)						
Global	0.23	1.36 (1.05–1.76)	0.020*			
Temporal	0.30	1.36 (1.05–1.76)	0.019*			
Temporal‐inferior	0.23	1.34 (1.03–1.73)	0.027*			
Nasal‐inferior	0.19	1.30 (1.02–1.67)	0.036*			
Nasal	0.20	1.35 (1.05–1.73)	0.021*			
Nasal‐superior	0.19	1.33 (1.03–1.71)	0.027*			
Temporal‐superior	0.25	1.35 (1.04–1.74)	0.023*			
PMB	0.30	1.36 (1.06–1.77)	0.018*			
NT ratio	0.09	0.85 (0.66–1.09)	0.212			

*Note*: All models were adjusted for age, sex, ethnicity, hypertension, cataract, diabetes, previous eye surgery, and disc area, with the exception of diabetes in the prevalent PD models (only 2 cases).

Abbreviations: PD, Parkinson's disease; SD, standard deviation of the mean; CI, confidence interval; PMB, papillomacular bundle; NT ratio, nasal‐temporal ratio.

* *P*‐value <0.05;

** *P*‐value <0.01.

### Associations between Optic Disc Pallor and PD Duration

In the prevalent PD group, and independent of covariates, each year since PD diagnosis was associated with a 1.37 SD increase in global pallor (standardized *β* = 1.37 [SE = 0.61], *P* = 0.029). The effect was strongest in the PMB zone (standardized *β* = 1.57 [SE = 0.62], *P* = 0.012), and present in the temporal (standardized *β* = 1.52 [SE = 0.62], *P* = 0.016) and nasal‐superior zones (standardized *β* = 1.46 [SE = 0.57], *P* = 0.013). In sensitivity analyses, removing participants with cataracts (n = 5) and then eye surgery (n = 5) reduced the strength of estimates and rendered the associations with global pallor non‐significant (significant associations with other zones were upheld). Removing participants with diabetes (n = 2) and non‐White ethnicity (n = 6) strengthened all the estimates. However, removing participants with PD duration >15 years (n = 5) rendered all associations non‐significant, indicating that the previously observed associations were strongly influenced by these five data points. There was no association between disc pallor and time to diagnosis in the incident PD group. Results are summarized in Table 3, and a visual representation showing the relationship between disc pallor and PD duration for the prevalent PD group is presented in Figure S3.

**TABLE 3:** Linear regression results for the association between optic disc pallor and years since diagnosis and years to diagnosis

Pallor	Years living with PD (prevalent PD group)			Years to diagnosis (incident PD group)		
	1 SD	*β* per SD increase (SE)	*P*‐value	1 SD	*β* per SD increase (SE)	*P*‐value
Global	0.25	1.37 (0.61)	0.029*		−0.11 (0.19)	0.565
Temporal	0.31	1.52 (0.62)	0.016*		−0.06 (0.19)	0.733
Temporal‐inferior	0.24	1.12 (0.62)	0.077		−0.17 (0.19)	0.366
Nasal‐inferior	0.21	1.02 (0.59)	0.088		−0.07 (0.18)	0.708
Nasal	0.22	1.07 (0.58)	0.071		−0.18 (0.18)	0.330
Nasal‐superior	0.22	1.46 (0.57)	0.013*		−0.20 (0.18)	0.272
Temporal‐superior	0.27	1.32 (0.61)	0.035*		−0.01 (0.19)	0.949
PMB	0.32	1.57 (0.62)	0.012*		−0.06 (0.19)	0.742
NT ratio	0.08	−1.06 (0.62)	0.089		−0.14 (0.17)	0.401
Sensitivity analysis						
Without cataract (N = 5 removed)						
Global	0.25	1.23 (0.65)	0.061			
Temporal	0.31	1.44 (0.65)	0.029*			
Nasal‐superior	0.22	1.31 (0.61)	0.035*			
PMB	0.31	1.50 (0.65)	0.024*			
Without eye surgery (N = 5 removed)						
Global	0.25	1.24 (0.65)	0.061			
Temporal	0.30	1.46 (0.65)	0.029*			
Nasal‐superior	0.22	1.31 (0.61)	0.035*			
PMB	0.31	1.51 (0.65)	0.023*			
Without diabetes (N = 2 removed)						
Global	0.25	1.56 (0.65)	0.018*			
Temporal	0.31	1.66 (0.64)	0.012*			
Nasal‐superior	0.22	1.64 (0.60)	0.008**			
PMB	0.32	1.71 (0.64)	0.009**			
White ethnicity only (N = 6 removed)						
Global	0.25	1.44 (0.66)	0.031*			
Temporal	0.30	1.60 (0.65)	0.016*			
Nasal‐superior	0.21	1.59 (0.64)	0.015*			
PMB	0.31	1.63 (0.64)	0.013*			
Without outliers (N = 5 removed with PD duration > 15 years)						
Global	0.25	0.50 (0.44)	0.260			
Temporal	0.31	0.60 (0.45)	0.185			
Nasal‐superior	0.21	0.57 (0.42)	0.178			
PMB	0.32	0.64 (0.45)	0.159			

*Note*: All models were adjusted for age, sex, ethnicity, hypertension, cataract, diabetes, previous eye surgery, and disc area, with the exception of diabetes in the prevalent PD models (only 2 cases). Significant *P*‐values in bold. *P‐value <0.05; **P‐value <0.01.

Abbreviations: PD, Parkinson's disease; SD, standard deviation of the mean; CI, confidence interval; PMB, papillomacular bundle; NT ratio, nasal‐temporal ratio.

## Discussion

We found that optic disc pallor was significantly higher in individuals with PD compared with controls, globally and across all zones. For each standard deviation increase in global optic disc pallor, the likelihood of being in the prevalent PD group compared to the age‐ and sex‐matched control group increased by 39%. Odds ratios were strongest temporally and weakest nasally, which is consistent with existing OCT studies in PD.^7^, ^17^ We also found that optic disc pallor increased with PD *duration* globally and across three zones. However, this association was strongly influenced by five individuals with PD duration of >15 years, so it should be interpreted with caution. These findings represent a step forward because, in comparison to OCT, color fundus photography is less expensive, quicker to acquire, more likely to be collected routinely (ie, during routine high‐street eye exams), and more directly correlates with clinical observation via slit‐lamp. Furthermore, fundus photography is widely accessible in both community and clinical settings, making it a practical tool for the potential monitoring of neurodegenerative conditions like PD.

Although the link between pRNFL thinning and PD is well established,^7^ research on the association between PD and optic disc pallor (which largely results from pRNFL thinning) is scant. One previous study reported an association^14^; however, its assessment of optic disc pallor was subjective and grouped under “optic nerve disorders” (including optic nerve head drusen, atrophy, and pallor) that were present in around two‐thirds of the 85 patients studied. By contrast, the current study uses quantitative measures of disc pallor from a larger sample and provides numerical estimates from statistical models that account for relevant covariates.

Previous work has used deep learning to investigate color fundus photographs for signs of PD. In one study utilizing the UK Biobank, a deep learning classifier was reported to distinguish PD from age‐ and sex‐matched controls with an accuracy of 68%, irrespective of incident and prevalent PD.^18^ Also using UK Biobank images, another study found that an individual's retinal age gap (retinal age minus chronological age) was associated with future risk of developing PD.^19^ A further study used color fundus photographs to predict the Hoehn and Yahr score, achieving an accuracy of between 65% and 75%.^20^ Although interesting and valuable, these studies arguably suffer from the “black box” problem inherent in deep learning‐based classifiers. Other than providing GradCAM images (heatmaps) that show “where” in an image the model is drawing inference from, they offer little clinically meaningful information. By contrast, optic disc pallor is a widely understood clinical sign in fundoscopic observation, indicating damage to the optic nerve and its pathways. Furthermore, our method uses segmentation and mathematical analysis, making the process transparent and understandable to clinicians and patients.

Although optic disc pallor is thought to be largely influenced by the number and health of the nerve fibers that course over the neuro‐retinal rim (eg, the pRNFL), it is possible that disc pallor could also result from a reduction in other retinal layers, such as the inner nuclear layer (INL) and the GCIPL, which contain the dopaminergic cells that are characteristically depleted in PD.^9^ In this respect, our results align with findings from a cross‐sectional analysis of the AlzEye cohort and the UK Biobank, which demonstrated that individuals with PD had thinner GCIPL and INL compared with controls.^5^ Optic disc pallor may also be influenced by perfusion. A recent meta‐analysis of nine OCT angiography studies found reduced macula perfusion density in PD patients compared with controls.^21^ Whereas OCT‐based studies emphasize retinal layer thinning and OCT angiography studies emphasize perfusion of the retinal microvasculature, our quantitative analysis of optic disc pallor may provide an additional, easily interpretable biomarker for PD. This complementary approach enriches the spectrum of retinal biomarkers available for PD assessment and highlights the multifaceted impact of PD on the retina. Further work should assess the unique association between optic disc pallor and retinal layers in a normal population.

Similar to retinal layer thickness,^22^ optic disc pallor has a wide distribution over the healthy population. In the current dataset of 787 participants, values of global pallor ranged from 0.63 to 2.0 (mean = 1.27, standard deviation = 0.23). As a result, establishing a universal cutoff value to identify patients with “high” optic disc pallor is challenging. This means that pallor values for a given individual may have limited prognostic value unless extreme. A better approach could be to measure the *rate‐of‐change* in pallor using consecutive images, thereby enabling the monitoring of PD progression and potentially facilitating the earlier detection of abnormal changes. Further work could assess if the rate‐of‐change in pallor correlates with other measures of symptoms.

In accordance with OCT findings, we observed that the effects of disc pallor in PD were slightly stronger in the temporal zones. This suggests that retinal changes in PD might reflect broader retinal neurodegeneration rather than isolated macular atrophy that leads to temporal pallor. Furthermore, current OCT studies indicate that macular changes in PD may be non‐linear, with perifoveal changes strongest superiorly and weakest temporally.^6^ Optic disc pallor may follow a similar pattern. Further work could test this hypothesis using concurrent OCT and fundus imaging data. However, the finding that temporal pallor has the strongest association with PD is probably not disease specific and instead likely reflects the underlying anatomy of the RNFL and its blood supply. The temporal aspect of the optic disc is generally more vulnerable to damage due to its thinner RNFL and less robust vascular network.^23^

In the current study, 22.8% of images were rejected for low quality, which is not unusual for color fundus photographs,^24^ especially given the age of the participants. Recent work has found that demographic factors can influence the likelihood that an image will be rejected for “low quality.” For example, images are more likely to be rejected if they are from certain demographic groups such as males, non‐White individuals, older adults, those with higher body mass index (BMI) or blood pressure, or those from the population of interest.^25^ In our study, we observed that more images were rejected from the control group (25%) compared to only 13.6% from the PD group, suggesting that our quality‐based exclusions did not disproportionately affect the PD group.

The associations between pallor and PD duration did not fully survive sensitivity analysis to cataracts and previous eye surgery. This suggests that these two factors may influence optic disc pallor and should be carefully controlled in future work. However, removing participants with diabetes *increased* model estimates, for both PD duration and logistic models assessing prevalent PD versus controls. This suggests that diabetes may act as a confounding factor, potentially masking the true relationship between optic disc pallor and PD. Individuals with diabetes have a higher risk of developing PD^26^; therefore, diabetes‐related changes in the eye (eg, diabetic retinopathy or other vascular complications) might obscure the effects of PD on optic nerve health. Future studies should carefully account for diabetes to better isolate the impact of PD on optic disc pallor.

The trend toward decreasing pallor with age (Fig. S4) may seem counterintuitive, especially because the RNFL typically thins with age.^27^ However, there are several potential explanations for this observation. As glial cells proliferate in response to chronic cellular stress, neurodegeneration, or vascular changes^28^—a process that intensifies with age—the optic nerve head's tissue density may increase.^29^ This could alter the reflective properties of the neuro‐retinal rim, making it appear darker and potentially reducing the observed pallor. Second, senile miosis, the age‐related decrease in pupil size, might also play a role. Smaller pupils reduce the amount of light and contrast in fundus images, potentially diminishing the apparent pallor scores for older individuals. Finally, because the pallor measure relies on relative contrast between the measurement and control region, localized changes to either of these will affect the result. These could include changes to the retinal pigment epithelium,^30^ lens opacity, refractive index, or the extracellular matrix and connective tissue composition of the optic nerve head. In short, although we believe pRNFL thickness remains the primary determinant of optic disc pallor, there is a complex interplay of various other age‐related factors that may influence the optic disc's appearance over time.

The strengths of this study include the use of novel software to mathematically quantify optic disc pallor in several zones, the relatively large sample size with the use of an age‐ and sex‐ matched control group, and the robust inclusion/exclusion criteria.

This study has some limitations. First, the associations between disc pallor and PD duration did not survive sensitivity analysis to outliers (removing five participants with PD duration >15 years rendered the associations non‐significant). This means that we must interpret this trend with caution. However, the five individuals with long PD duration are valid data points with images that passed all quality control checks; therefore, these data highlight the need for further investigation to better understand the potential effects of long‐term PD on the retina. Second, pallor varied substantially across different ethnicities (Fig. S1), but due to the limited number of participants in each ethnic category, we were unable to fully account for this heterogeneity in our statistical models. Third, *PallorMetrics* can be either fully‐ or semi‐automated. In the current study, we opted for the semi‐automated version to minimize processing error and retain as many participants/images as possible. This is both a strength in the sense that more images were successfully processed, and measurement error was minimized, and a limitation in that the results may not be precisely replicable. However, we point to an inter‐annotator agreement score of 94.2% in previous work^10^ that demonstrates that the methodology is replicable in our hands. Fourth, we relied on ICD‐10 codes for diagnostic definitions, which may not capture cases coded in outpatient clinics, thereby leading to case under‐ascertainment. Fifth, the effect sizes are relatively small, and replication in a larger, more diverse cohort is required. Sixth, UK Biobank data does not currently allow us to explore PD phenotype or severity, limiting our ability to further stratify the groups.

## Conclusion

This study revealed significant associations between optic disc pallor in color fundus photographs and PD, with the temporal zones showing the strongest effect. Pallor severity also correlated with PD duration, although this effect was strongly influenced by five individuals with long PD duration, so it should be interpreted cautiously. Our findings highlight the potential of optic disc pallor as a biomarker complementary to OCT measures for assessing PD‐related neurodegeneration. Future research should focus on longitudinal assessments (including in a prodromal PD population) and include a more ethnically diverse sample to enhance the generalizability of these findings.

## Author Roles

1) Research project: A. Conception, B. Organization, C. Execution; 2) Statistical Analysis: A. Design, B. Execution, C. Review and Critique; 3) Manuscript Preparation: A. Writing of the first draft, B. Review and Critique.

SG: 1A, 1B, 1C, 2A, 2B, 3A

DPB: 2C, 3C

TJM: 2C, 3C

## Full financial disclosures for the previous 12 months

S.G. has no conflicts of interest to report. T.J.M, is a founder, non‐executive director, and scientific advisor at Eye to the Future Limited. D.P.B has received project grants from the Reta Lila Weston Trust and the Mary Kinross Charitable Trust and honoraria from Bial Pharma UK Ltd and Springer Nature Limited. The authors report no conflicts of interest.

## Supporting information


**Figure S1.** Boxplot of global pallor by ethnicity for the entire sample (N = 787). Black diamonds represent the mean.
**Figure S2.** Examples of rejected images. (**A**) Under exposure, (**B**) over exposure, (**C**) uneven illumination around the optic disc, (**D**) abnormal presentation of retina, and (**E**) disc margin unclear.
**Figure S3.** Scatterplot showing the relationship between temporal pallor and years since the diagnosis for prevalent PD cases.
**Figure S4.** Boxplot of global pallor by age for the entire sample (N = 787).
**Figure S5.** Boxplots showing temporal pallor by prevalent and incident PD compared with controls.

## Data Availability

This research was conducted using data from the UK Biobank under project ID 95450. Data directly supporting the results of this work are available only to the immediate research team members due to UK Biobank's access control policy. Bona fide researchers can, however, apply for access at ukbiobank.ac.uk/enable-your-research/apply-for-access. UK Biobank data are available for approved researchers via successful application (https://www.ukbiobank.ac.uk/enable-your-research/apply-for-access). Retinal variables and processed images generated for the current study will be made available to UK Biobank approved researchers upon completion of this project. The *PallorMetrics* software is available upon request from the lead author.
