# Supplementary material for: Optic Disc Pallor in Parkinson's Disease: A UK Biobank Study
Source: Mov Disord. 2025 Jan 30;40(6):1123–33. doi: 10.1002/mds.30127 (PMC12160988; doi:10.1002/mds.30127)
Supplement: Supplementary file 1 — Figure S1. Boxplot of global pallor by ethnicity for the entire sample (N = 787). Black diamonds represent the mean. Figure S2. Examples of rejected images. (A) Under exposure, (B) over exposure, (C) uneven illumination around the optic disc, (D) abnormal presentation of retina, and (E) disc margin unclear. Figure S3. Scatterplot showing the relationship between temporal pallor and years since the diagnosis for prevalent PD cases. Figure S4. Boxplot of global pallor by age for the entire sample (N = 787). Figure S5. Boxplots showing temporal pallor by prevalent and incident PD compared with controls. [file MDS-40-1123-s001.docx]

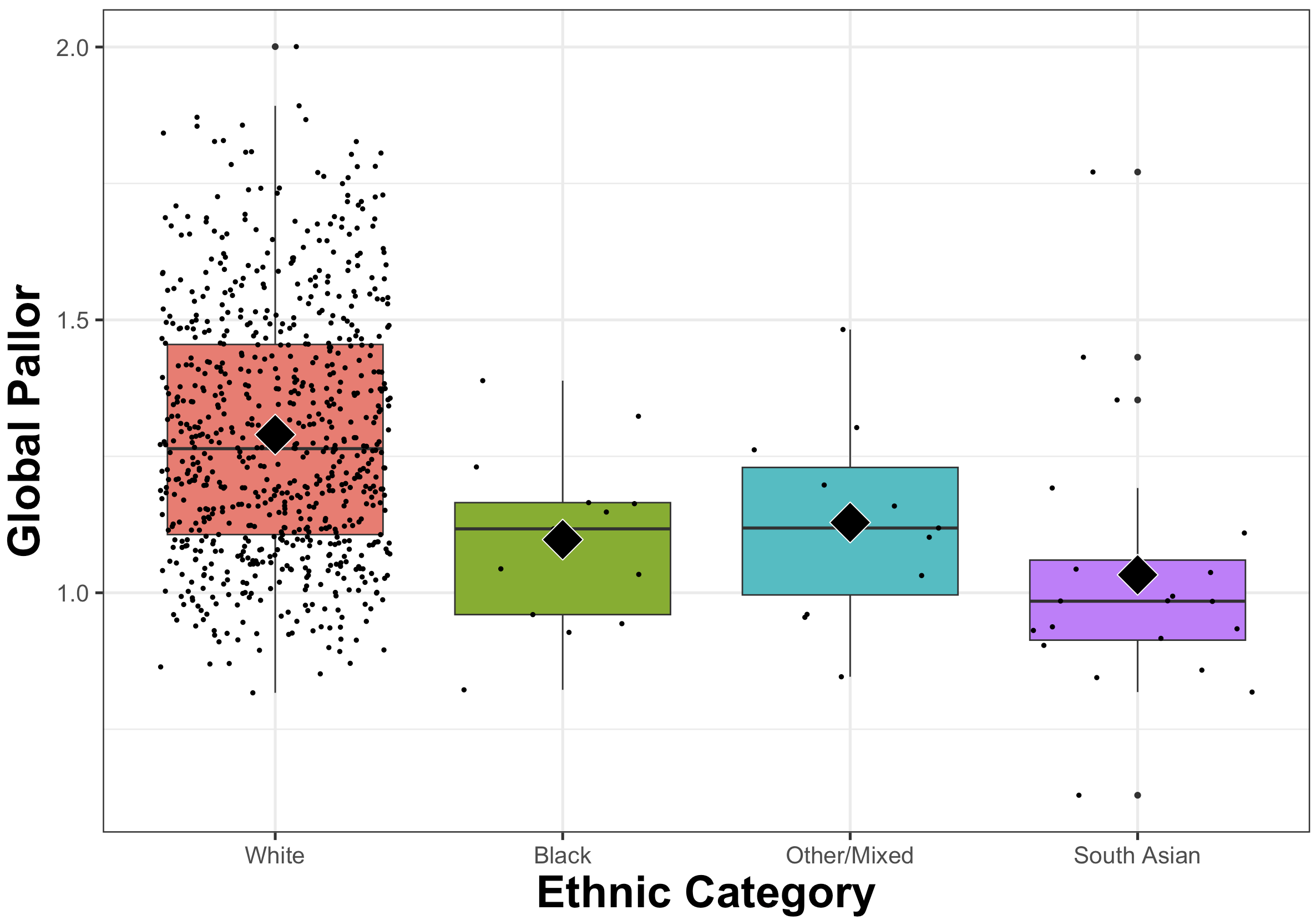
**Supplementary Figure 1.** Boxplot of global pallor by ethnicity for the entire sample (N = 787). Black diamonds represent the mean.


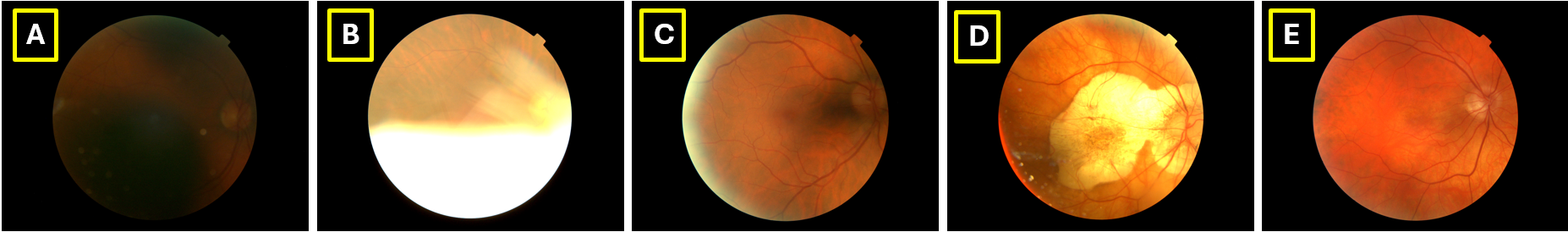


**Supplementary Figure 2.** Examples of rejected images. A) Under exposure, B) over exposure, C) uneven illumination around the optic disc, D) abnormal presentation of retina, E) disc margin unclear.


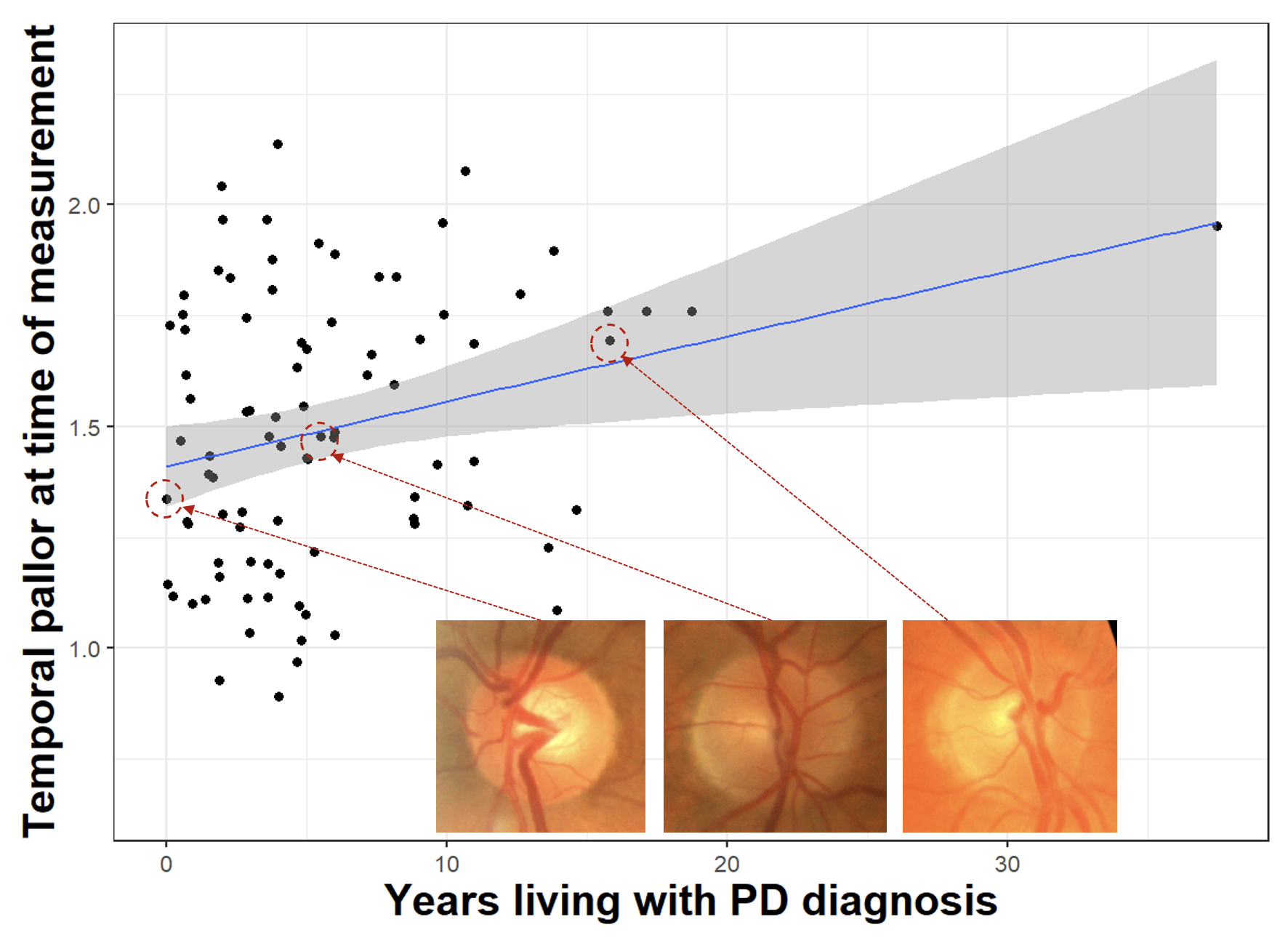


**Supplementary Figure 3**. Scatterplot showing the relationship between temporal pallor and years since diagnosis for prevalent PD cases.


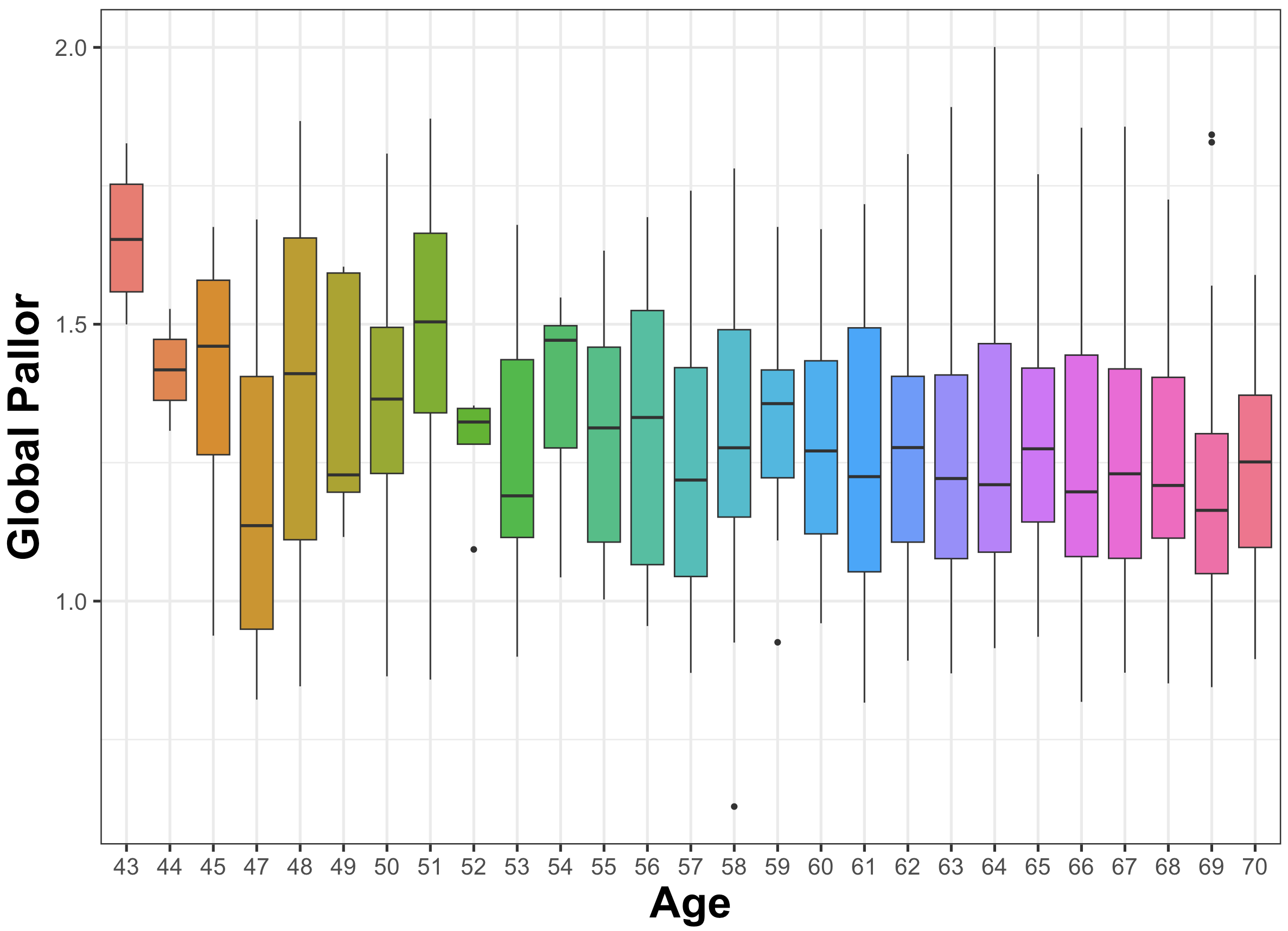


**Supplementary Figure 4.** Boxplot of global pallor by age for the entire sample (N = 787).


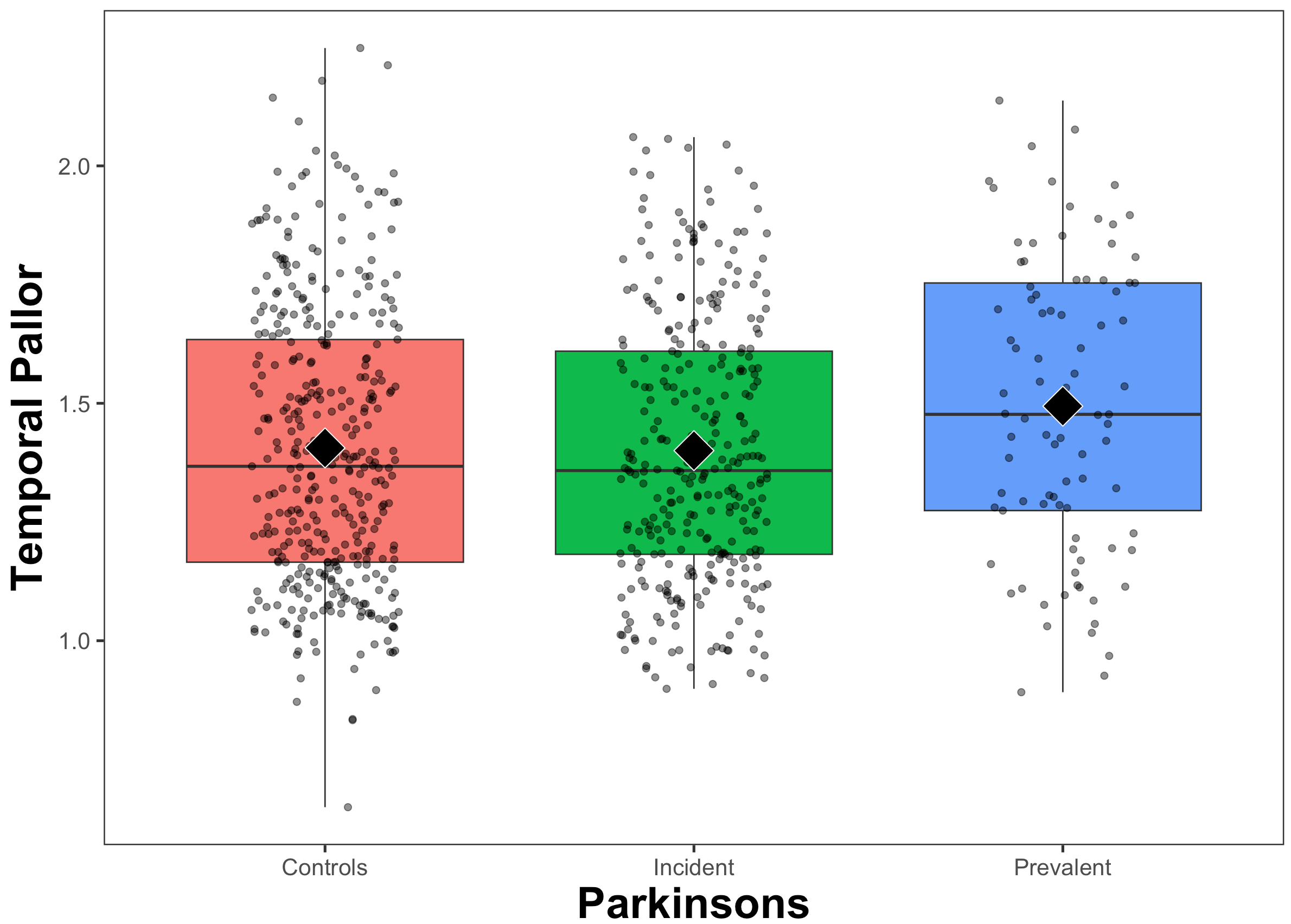


**Supplementary Figure 5**. Boxplots showing temporal pallor by prevalent and incident PD compared with controls.
